# Kras^G12D^ upregulates Notch signaling to induce gallbladder tumorigenesis in mice

**DOI:** 10.18632/oncoscience.368

**Published:** 2017-10-23

**Authors:** Wen-Cheng Chung, Junqing Wang, Yunyun Zhou, Keli Xu

**Affiliations:** ^1^ Cancer Institute, University of Mississippi Medical Center, Jackson, MS, USA; ^2^ Department of Surgery, Ruijin Hospital, Shanghai Jiao Tong University School of Medicine, Shanghai, China; ^3^ Department of Data Science, University of Mississippi Medical Center, Jackson, MS, USA; ^4^ Department of Neurobiology and Anatomical Sciences, University of Mississippi Medical Center, Jackson, MS, USA

**Keywords:** gallbladder tumorigenesis, Kras mutation, Notch signaling, mouse model, adenoma

## Abstract

**Background:**

Kras mutations and increased Notch activation occur frequently in gallbladder cancer. However, their roles in gallbladder carcinogenesis have not been defined. This study was aimed at determining whether expression of mutant Kras was sufficient to induce gallbladder carcinoma and whether Notch deregulation played a role in this context.

**Methods:**

We determined Cre recombination activity of *Pdx1-Cre* in the gallbladder using a reporter strain and examined gallbladder tumor development in the *Kras^LSL- G12D/+^;Pdx1-Cre* mice. We analyzed expression of Notch pathway genes in the mouse gallbladder by immunohistochemistry, quantitative RT-PCR, and Western blot analysis. We also determined the effect of *Jag1* deletion on Kras-induced gallbladder tumor development.

**Results:**

*Pdx1-Cre* exhibits robust recombination activity in the gallbladder epithelium. *Kras^LSL-G12D/+^;Pdx1-Cre* mice form early onset adenoma in the gallbladder and adjacent biliary tract with complete penetrance, albeit short of invasive adenocarcinoma. Kras^G12D^ upregulates expressions of *Notch2, Notch3, Notch4*, *Jag1* and downstream target genes *Hes1*, *Hey1* and *Hey2*, and deletion of *Jag1* partially suppresses Kras^G12D^-induced adenoma development.

**Conclusions:**

Kras^G12D^ induces gallbladder adenoma and Notch plays a key role in Kras-initiated gallbladder tumorigenesis.

## INTRODUCTION

Gallbladder cancer is the most common malignancy of the biliary tract, associated with late diagnosis, unsatisfactory treatment and poor prognosis. There are two key pathways leading to gallbladder carcinogenesis. The fi involves gallstones and the resultant cholecystitis. The second involves congenital anomalous pancreatobiliary duct junction (APBDJ). Gallbladder cancers evolved through these two pathways demonstrate differences in demographic distribution, clinical outcome, gender bias and molecular changes. Regarding the molecular changes, KRAS mutations are frequent and early events in tumors associated with APBDJ, but less frequent in carcinomas associated with gallstones [1]. Other molecular changes of gallbladder cancers include somatic mutations of TP53, ErbB signaling pathway genes, and cell cycle regulator CDKN2A [2, 3]. Overexpression of Erbb2 in the basal epithelium of the mouse gallbladder resulted in the development of adenoma and progression to adenocarcinoma characterized by papillary structures, demonstrating a functional role of Erbb2 in gallbladder carcinogenesis [4]. No comprehensive analysis for other molecular changes has been carried out using *in vivo* model system.

Notch signaling controls cell fate determination, cell differentiation and proliferation in a variety of tissues during development as well as in adult homeostasis. Haploinsuffi for the Notch ligand JAG1 results in an autosomal-dominant, multisystem disorder known as Alagille syndrome, which is characterized by a congenital cholangiopathy of variable severity. Similarly, Jag1 heterozygosity in mice results in impaired intrahepatic bile duct development [5]. Notch2 appears to interact with Jag1 in the pathogenesis of Alagille syndrome [6]. Conversely, high level expression of Notch pathway genes has been found in human gallbladder cancer, and Notch 1 and Notch 3 expression is correlated with severe clinicopathological characteristics and poor prognosis [7–9]. However, the mechanism of Notch deregulation and its role in gallbladder cancer initiation and progression remain unclear.

In the present study, we determined the outcome of expression of the most common form of Kras mutations (KrasG12D) in the mouse gallbladder epithelium. We also determined whether Notch signaling is deregulated by KrasG12D and contributes to the gallbladder tumorigenesis.

## RESULTS

### Expression of Kras^G12D^ in the gallbladder epithe- lial cells induces adenoma

**Figure 1:**
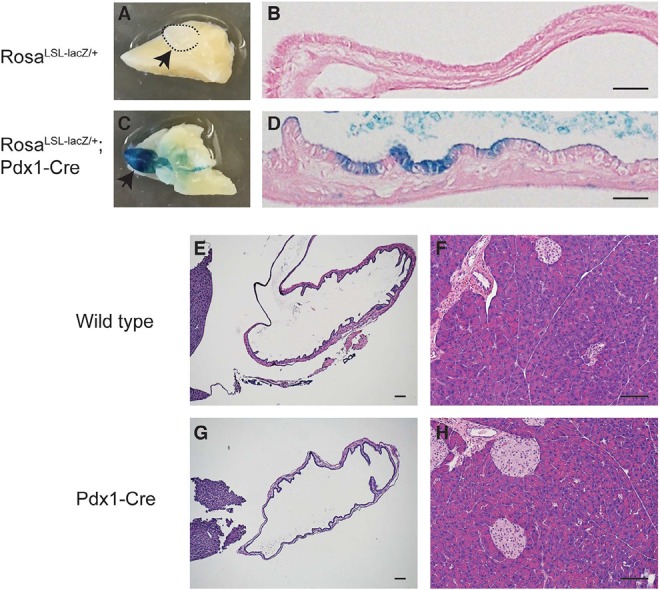
*Pdx1* promoter drives Cre expression in the gallbladder epithelium (**A-D**) Representative photographs of X-Gal staining of the gallbladder from *Rosa^LSL-lacZ/+^* and *Rosa^LSL-lacZ/+^;Pdx1-Cre* mice at 5 weeks of age (left: whole-mount; right: sectioned). **(E-H)** Representative photographs of H&E-stained sections of the gallbladder (left panels) and pancreas (right panels) from wild type and *Pdx1- Cre* mice at 4 months of age. Scale bars: 20 μm in B and D; 50 μm in E-H.

Pdx1 is known to be expressed in endocrine and some duct cells and may identify a more primitive population in the adult pancreas. Interestingly some epithelial cells in adult human gallbladder highly express Pdx1 [10]. We crossed a *Rosa^LSL-lacZ^* reporter into the *Pdx1- Cre* mouse and performed X-gal staining in the gallbladder to determine the Cre recombination activity. Robust LacZ activity was observed in a subset of ductal epithelial cells in *Rosa^LSL-lacZ/+^;Pdx1-Cre* gallbladder, but not in *Rosa^LSL- lacZ+^* control (Figure 1A-1D). Therefore, *Pdx1-Cre* can be used to drive expression of Kras^G12D^ in the gallbladder, in addition to the pancreas as previously reported [11]. Of note, *Pdx1-Cre* mice undergo normal development of the gallbladder and pancreas (Figure 1E-1H).

**Figure 2:**
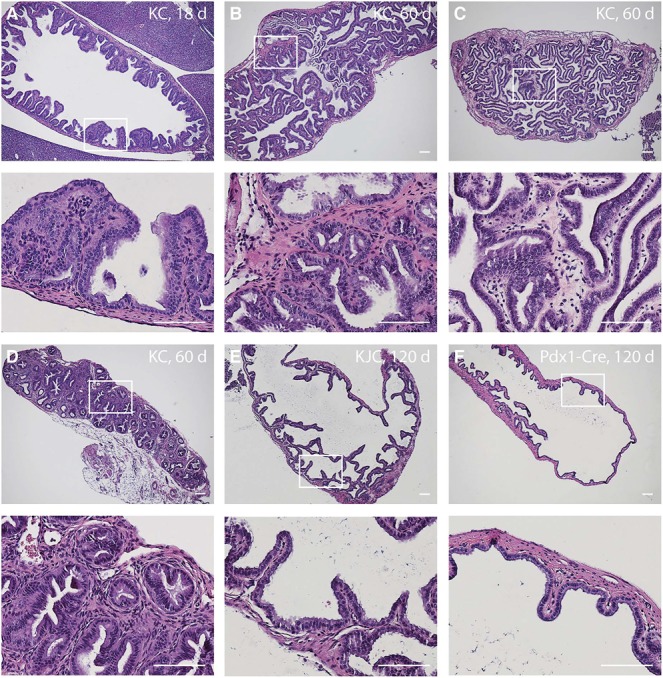
Expression of Kras^G12D^ in the gallbladder epithelium using Pdx1-Cre induces adenoma and low-grade adenocarcinoma (**A-D**) Representative photographs of H&E-stained gallbladder sections from KC mice of indicated age. **(E)** H&E- stained gallbladder section of a KJC mouse at 120 days. **(F)** H&E staining of the *Pdx1-Cre* gallbladder at 120 days. High magnification images are shown below each panel. Scale bars: 50 μm.

We examined tumor development in the gallbladder of *Kras^LSL-G12D/+^;Pdx1-Cre* (KC) mice. As early as 18 days after birth, KC mice showed gallbladder epithelial hyperplasia (Figure 2A). By 2 months of age, all animals examined (10/10) developed gallbladder adenoma prior to the detection of signifi lesion in the pancreas. These tumors are composed of papillary structures lined by cuboidal or columnar epithelial cells with moderate atypia (Figure 2B, 2C) or tubular glands (Figure 2D). It is noteworthy that papillary adenomas were prevalent in the gallbladder while tubular glandular lesions occurred more frequently in the adjacent biliary tract. No invasive adenocarcinoma was found in KC mice up to 5 months of age. The histology of the *Pdx1-Cre* gallbladder is completely normal (Figure 2F).

### Expression of Kras^G12D^ causes upregulation of Notch receptors and Jag1 ligand and increased Notch signaling in the gallbladder

**Figure 3:**
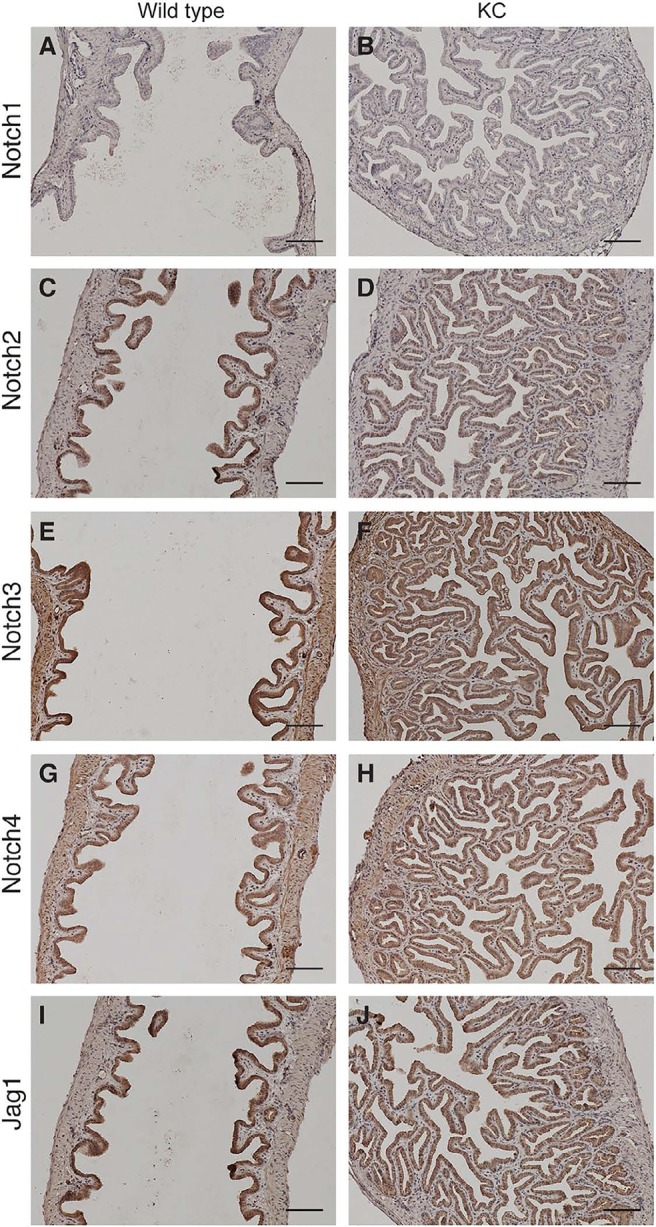
Expressions of Notch receptors and Jag1 ligand in the mouse gallbladder Shown are representative photographs of immunostaining for Notch receptors and Jag1 ligand in the gallbladder of wild type and KC mice at 2 months of age. Scale bars: 50 μm.

**Figure 4:**
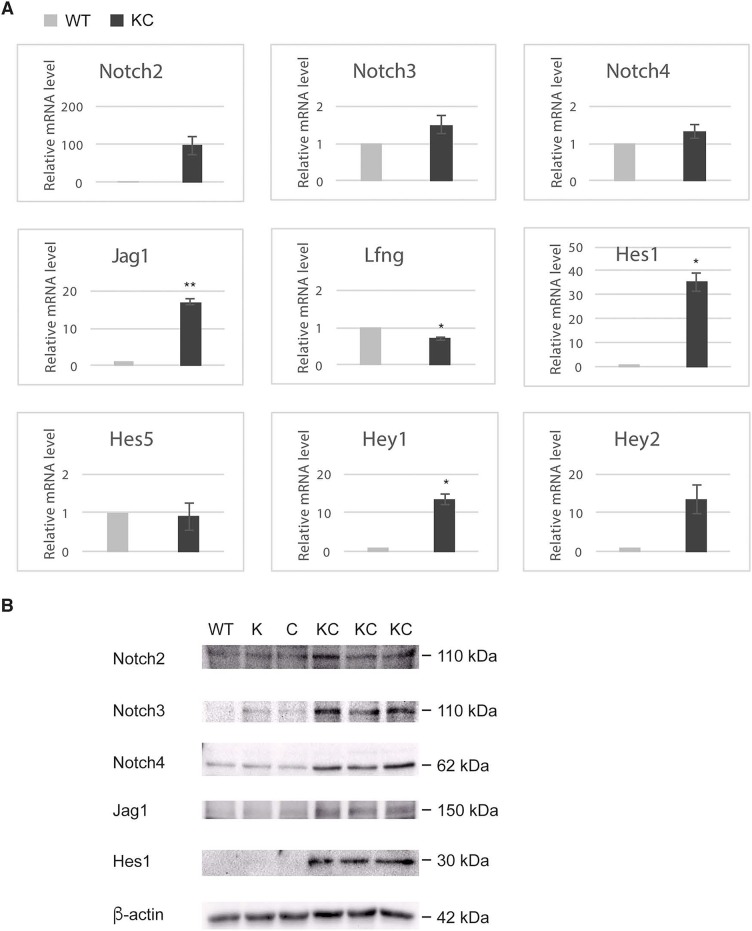
Upregulation of Notch signaling pathway in the gallbladder of KC mice **(A)** Quantitative RT-PCR for Notch pathway genes in the gallbladder of wild type and KC mice at 1 month of age. *p < 0.05, **p < 0.005. **(B)** Western blot analysis for Notch pathway genes in the gallbladder of indicated genotype at 40 days of age. K: *Kras^LSL-G12D/+^*; C: *Pdx1-Cre*.

Aberrant expression of Notch receptors has been suggested to play a role in extrahepatic cholangiocarcinoma and gallbladder carcinoma [8]. We performed immunohistochemistry of Notch receptors and Jag1 ligand on the gallbladder sections at 2 months of age. Both wild type and KC mice showed intense staining of Notch2, Notch3, Notch4 and Jag1 in the gallbladder epithelial cells, whereas Notch1 immunoreactivity was very weak or undetectable (Figure 3). We determined the mRNA levels of Notch pathway genes in the gallbladder by quantitative RT-PCR. Transcripts of Notch2 and Jag1 were increased 97- and 17-fold, respectively, while Notch3 and Notch4 mRNA levels were slightly increased in KC as compared to the wild type (Figure 4A). The expression of Lfng, which is known to inhibit Jagged/Serrate-mediated Notch activation [12], was downregulated in KC mice. Transcription of Notch downstream target genes *Hes1*, *Hey1*, and *Hey2* were all increased drastically in KC mice (Figure 4A). Next, we performed Western blot analysis for Notch pathway components in the gallbladder. KC mice showed consistent increase of the protein levels of Notch2, Notch3, Notch4, Jag1, and Hes1 as compared to control mice including the wild type, *Kras^LSL-G12D/+^* and *Pdx1-Cre* (Figure 4B). Taken together, Notch signaling is upregulated by the expression of Kras^G12D^ in the gallbladder epithelial cells. Interestingly, 4 out of 8 *Kras^LSL-G12D/+^;Jag1^flox/flox^ ;Pdx1-Cre* (KJC) mice between 4 and 6 months of age showed near normal histology of the gallbladder, suggesting that deletion of *Jag1* partially suppresses Kras^G12D^-induced adenoma development (Figure 2E).

### Expression of Notch pathway genes in the human gallbladder

**Figure 5:**
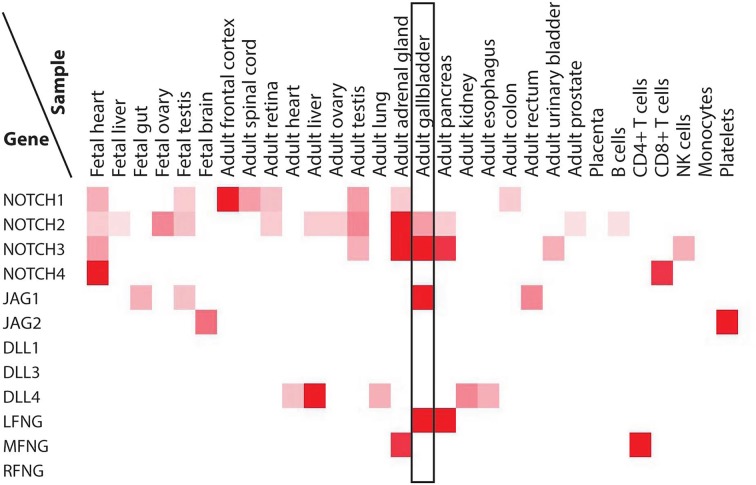
Protein levels of Notch receptors, ligands, and Fringes in various human tissues Data were derived from http://www.humanproteomemap.org.

We surveyed protein levels of Notch receptors, ligands, and Fringes in human tissues using published proteomic data (http://www.humanproteomemap.org) [13]. As shown in Figure 5, expressions of NOTCH2, NOTCH3, JAG1 and LFNG are enriched in the adult gallbladder. This is in agreement with our analysis of Notch pathway components in murine gallbladder, suggesting that JAG1-mediated, LFNG-modulated NOTCH2/3 signaling may well play an important role in KRAS-initiated gallbladder carcinogenesis in humans.

## DISCUSSION

In a model for multistage pathogenesis of the most common form of gallbladder cancer beginning with gallstones and chronic cholecystitis, Kras mutations are thought to occur at the very late stage [3]. On the other hand, previous study also found higher frequency of Kras mutations in gallbladder adenomas compared with carcinomas, hinting that gallbladder adenomas and carcinomas may arise through distinct molecular pathways [14]. In the present study, the expression of KrasG12D caused papillary adenoma as early as one month of age (data not shown). It is interesting that Kras mutations were found more frequently in patients with papillary adenocarcinoma compared with other types of adenocarcinoma or squamous cell carcinoma [15]. However, early onset adenomas in KC mice do not progress into invasive carcinoma, suggesting additional molecular changes may be required for the development of adenocarcinoma.

Notch signaling pathway plays a central role in the development of biliary system. Hes1-deficient mice exhibit gallbladder agenesis and severe hypoplasia of extrahepatic bile ducts [16]. Double heterozygous mice of Jag1 and Notch2 display jaundice associated with defects in bile duct epithelial cell differentiation and morphogenesis [6]. Here we show that expression of KrasG12D upregulates Notch gene expression, suggesting that Kras may induce gallbladder adenoma through activation of Notch signaling. Hes1 is the most upregulated gene among canonical Notch downstream target genes in the KC gallbladder. Given that loss-of-Hes1 results in gallbladder agenesis, we speculate that Hes1 controls gallbladder epithelial cell proliferation and/or differentiation. Lfng, a negative regulator of Serrate/ Jagged-mediated Notch signaling, is highly expressed in the adult human gallbladder. Kras^G12D^ downregulates Lfng expression in the gallbladder, raising the possibility that Lfng normally inhibits Notch activation to prevent gallbladder adenoma formation. It is also likely that Kras- mediated Notch deregulation promotes tumor development in a subset of gallbladder cancer patients, and targeting Notch2/3 and Jag1 could be a therapeutic approach for these patients.

Understanding of gallbladder carcinogenesis is limited in part due to the paucity of animal models for this disease. One model is the overexpression of ErbB2 in the basal layer of biliary tract epithelium under the control of the bovine keratin 5 promoter [4]. Another involves the inactivation of the oxysterol receptor liver X receptor–β in female mice, resulting in preneoplastic lesions of the gallbladder and progression to cancer in old animals [17]. We have shown that Pdx1 promoter is active in the gallbladder epithelium. Thus, forced expression or inactivation of genes in the gallbladder using Pdx1-Cre can be a new approach in developing mouse models for gallbladder cancer.

## MATERIALS AND METHODS

### Mice

*Kras^LSL-G12D/+^*, *Rosa^LSL-lacZ/+^* and *Pdx1-Cre* mouse strains were obtained from the Jackson Laboratory and have been previously described [18–20]. *Jag1^flox^* strain was provided by Dr. Radtke and described previously [21]. Mouse experiments were performed in accordance with a protocol approved by the Institutional Animal Care and Use Committee of the University of Mississippi Medical Center.

### Histology, immunohistochemistry, X-Gal staining, and western blot

Formalin-fixed paraffin-embedded gallbladder tissues were processed for histology and immunohistochemistry by standard procedures. Primary antibodies used for immunostaining were: Notch1 (Cell Signaling, No. 3608, 1:100), Notch2 (DSHB, University of Iowa, C651.6DbHN, 1:200), Notch3 (ProteinTech, 55114-1-AP, 1:100), Notch4 (Millipore, 09-089, 1:100), and Jagged1 (Santa Cruz, sc-6011, 1:100). X-Gal staining was performed as previously described [22]. For Western blot analysis, gallbladder tissues were lysed in RIPA buffer (Boston BioProducts) supplemented with protease inhibitor (Roche), and processed according to standard methodology. Antibodies for probing specified proteins are as follows: Notch1, Notch2, Notch3, Notch4 and Jagged1 are the same as above (all with 1:1000 dilution), Hes1 (Millipore, AB5702, 1:500), and β-Actin (Santa Cruz, sc-81178, 1:1000).

### Quantitative RT-PCR

Total RNA was extracted from the gallbladder using RNeasy Mini kit (Qiagen) and reverse-transcribed using iScript cDNA synthesis kit (Bio-rad). PCR was performed using QuantiTect SYBR Green PCR Kits (Qiagen) with BioRad CFX96 qPCR System. Results were normalized with the expression level of Gapdh and presented as fold change of the control. The experiment was performed in triplicate and presented as mean ± standard error. Unpaired two-tailed t-test was performed for comparison between the mutant and wild type mice. Primer sequences for *Jag1*, *Lfng*, *Hes1*, *Hey1* and *Hey2* have previously been reported [11, 23]. Other primer sequences are as follows: Notch2, 5′-AACTGTCAGACCCTGGTGAAC-3′ (forward), 5′-CGACAAGTGTAGCCTCCAATC-3′ (reverse); Notch3, 5′-TGCCAGAGTTCAGTGGTGG-3′ (forward), 5′-CACAGGCAAATCGGCCATC-3′ (reverse); Notch4, 5′-AGGGAGGCTCTGTGAGGTGG-3′ (forward), 5′-ATCCAGGAAGGCAGAGGCAC-3′ (reverse).

## Abbreviations

APBDJ: anomalous pancreatobiliary duct junction
KC: *Kras^LSL-G12D/+^;Pdx1-Cre*
KJC: *Kras^LSL-G12D/+^;Jag1^flox/flox^;Pdx1-Cre*
